# Supplementary material for: Is mHealth a Useful Tool for Self-Assessment and Rehabilitation of People with Multiple Sclerosis? A Systematic Review
Source: Brain Sci. 2021 Sep 9;11(9):1187. doi: 10.3390/brainsci11091187 (PMC8466296; doi:10.3390/brainsci11091187)
Supplement: Supplementary file 1 [file brainsci-11-01187-s001.zip › brainsci-1338516-supplementary.pdf]

Table S1: Search strategy for PubMed

	MeSH term	Hits
#1 population	(multiple sclerosis) OR (ms) OR (multiple sclerosis[MeSH Terms])	510,099
#2 mHealth tool	(ehealth[MeSH Terms]) OR (ehealth) OR (mhealth) OR (mobile apps) OR (smartphone applications) OR (apps)	68,807
#3 disease management	(self-monitoring) OR (self- assessment) OR (functioning) OR (intervention) OR (rehabilitation)	19,493,401
#1 AND #2 and #3	((multiple sclerosis) OR (ms) OR (multiple sclerosis[MeSH Terms])) AND ((ehealth[MeSH Terms]) OR (ehealth) OR (mhealth) OR (mobile apps) OR (smartphone applications) OR (apps)) AND ((self-monitoring) OR (self-assessment) OR (functioning) OR (intervention) OR (rehabilitation))	1,063

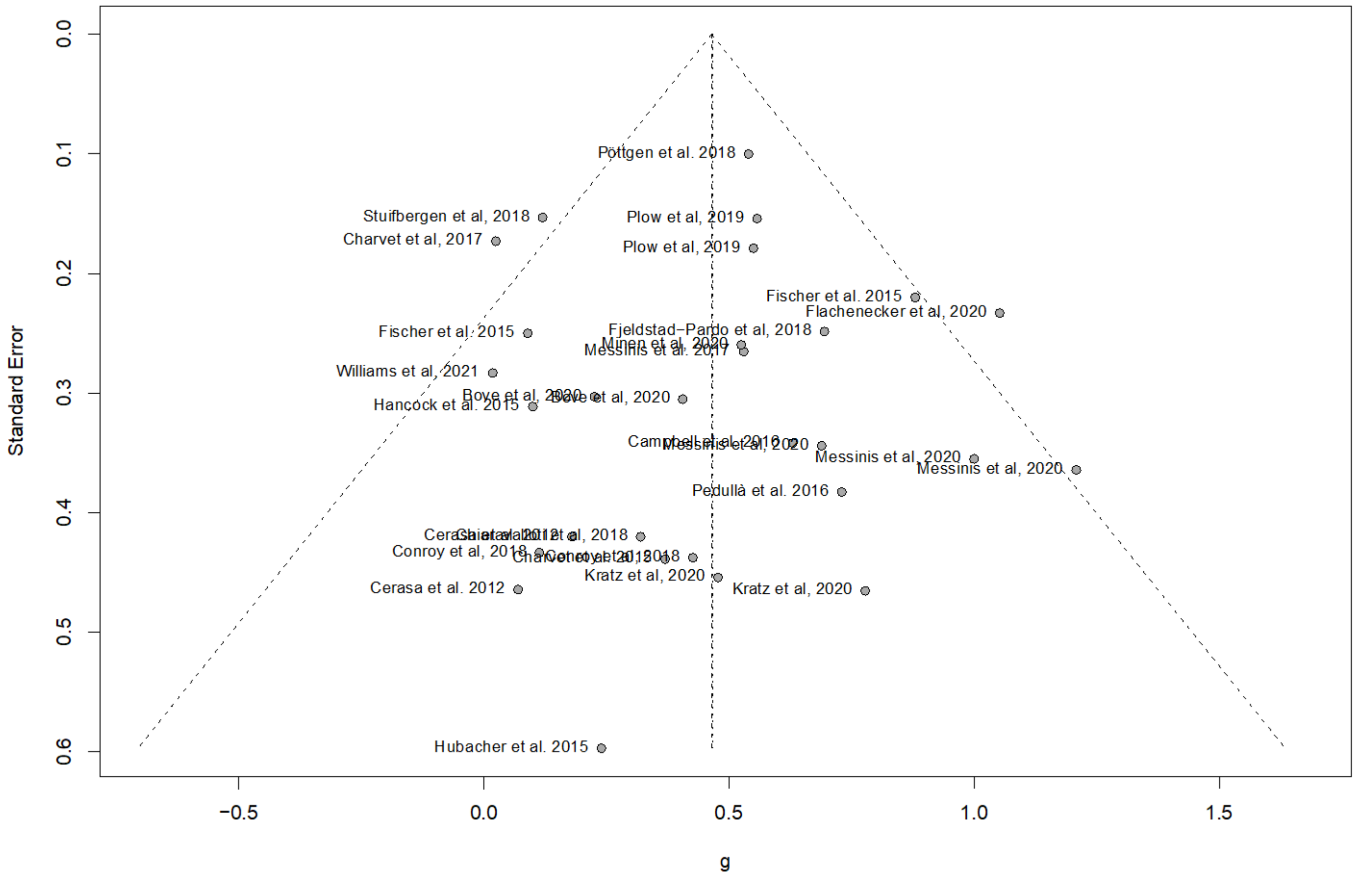

Figure S1. Funnel plot of the included studies in the meta-analysis

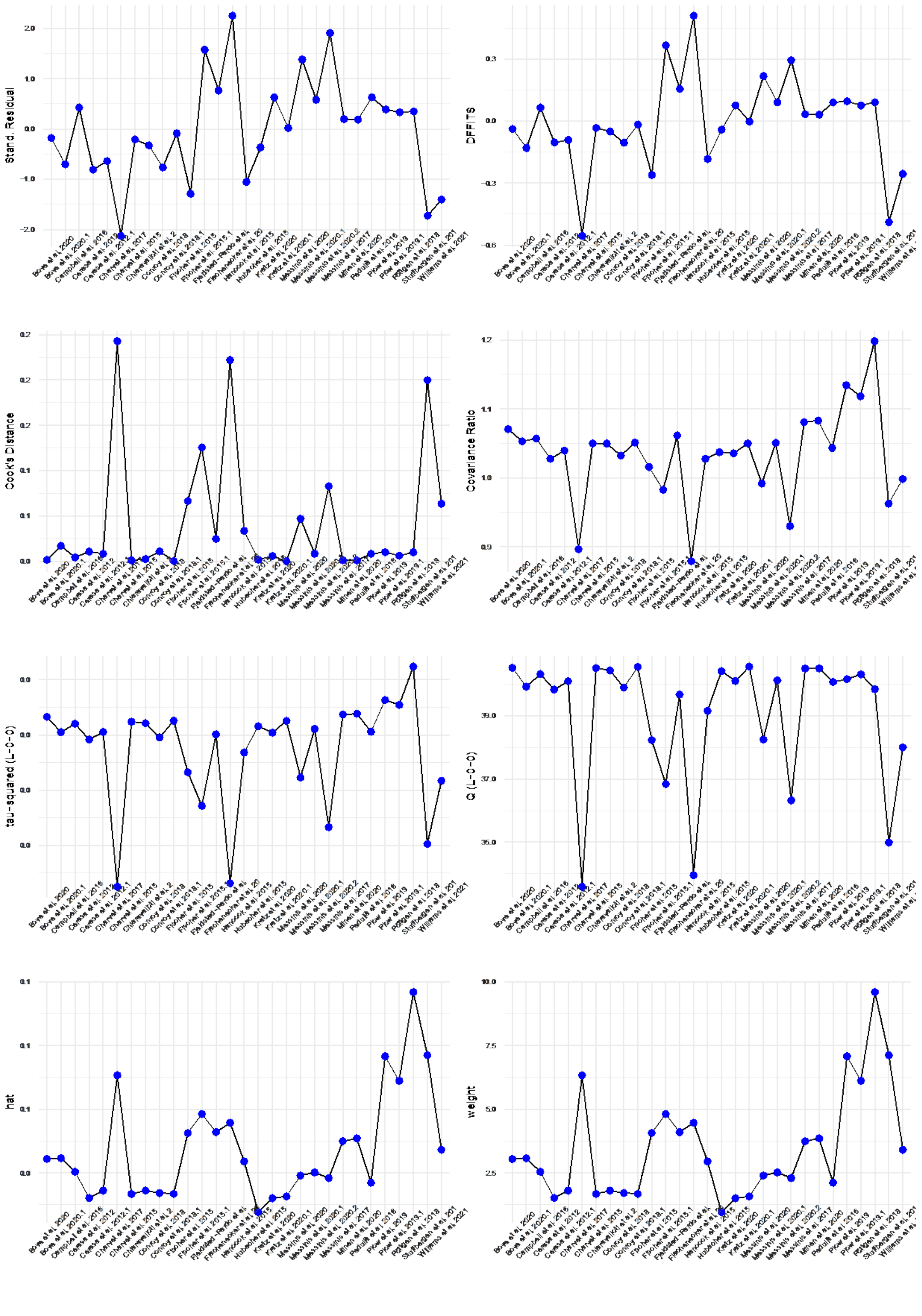

Figure S2. Sensitivity analysis of the included studies in the meta-analysis
